# Research Progress on Stem Cell Therapies for Articular Cartilage Regeneration

**DOI:** 10.1155/2021/8882505

**Published:** 2021-02-12

**Authors:** Shuangpeng Jiang, Guangzhao Tian, Xu Li, Zhen Yang, Fuxin Wang, Zhuang Tian, Bo Huang, Fu Wei, Kangkang Zha, Zhiqiang Sun, Xiang Sui, Shuyun Liu, Weimin Guo, Quanyi Guo

**Affiliations:** ^1^Institute of Orthopedics, The First Medical Center, Chinese PLA General Hospital, Beijing Key Lab of Regenerative Medicine in Orthopedics, Key Laboratory of Musculoskeletal Trauma and War Injuries PLA, No. 28 Fuxing Road, Haidian District, Beijing 100853, China; ^2^Department of Orthopedics, The First Hospital of China Medical University, 155 Nanjing North Street, Heping District, Shenyang 110001 Liaoning Province, China; ^3^School of Medicine, Nankai University, Tianjin 300071, China; ^4^Department of Orthopedic Surgery, First Affiliated Hospital, Sun Yat-sen University, Guangzhou, Guangdong, China

## Abstract

Injury of articular cartilage can cause osteoarthritis and seriously affect the physical and mental health of patients. Unfortunately, current surgical treatment techniques that are commonly used in the clinic cannot regenerate articular cartilage. Regenerative medicine involving stem cells has entered a new stage and is considered the most promising way to regenerate articular cartilage. In terms of theories on the mechanism, it was thought that stem cell-mediated articular cartilage regeneration was achieved through the directional differentiation of stem cells into chondrocytes. However, recent evidence has shown that the stem cell secretome plays an important role in biological processes such as the immune response, inflammation regulation, and drug delivery. At the same time, the stem cell secretome can effectively mediate the process of tissue regeneration. This new theory has attributed the therapeutic effect of stem cells to their paracrine effects. The application of stem cells is not limited to exogenous stem cell transplantation. Endogenous stem cell homing and in situ regeneration strategies have received extensive attention. The application of stem cell derivatives, such as conditioned media, extracellular vesicles, and extracellular matrix, is an extension of stem cell paracrine theory. On the other hand, stem cell pretreatment strategies have also shown promising therapeutic effects. This article will systematically review the latest developments in these areas, summarize challenges in articular cartilage regeneration strategies involving stem cells, and describe prospects for future development.

## 1. Introduction

Articular cartilage is an important weight-bearing tissue of synovial joints. Due to the lack of blood vessels, nerves, and lymphatic vessels and the restriction of the dense extracellular matrix (ECM) on cartilage cells, the self-healing ability of articular cartilage after injury is very limited. If left untreated, damage to articular cartilage can lead to osteoarthritis (OA) [1]. OA has a high incidence and disability rate, affecting 250 million patients worldwide [2]. Unfortunately, none of the cartilage repair techniques currently in clinical use can completely regenerate hyaline cartilage [3].

Stem cells are an important milestone in the field of tissue engineering and regenerative medicine. Stem cell therapy is considered to be a promising method to solve the regeneration of articular cartilage [4, 5]. A large number of preclinical and clinical studies have shown that compared with traditional repair techniques such as microfractures, stem cell therapy can form more typical hyaline cartilage and can better control symptoms [6–8]. On the other hand, compared with autologous chondrocytes, stem cells have a wider source and stronger ability to expand *in vitro*, which makes tissue-engineered cartilage involving stem cells more advantageous than tissue-engineered cartilage involving autologous chondrocytes. Tissue engineering strategies involving stem cells involve the implantation of exogenous stem cells and homing of endogenous stem cells to achieve cartilage regeneration in situ. The basis of the exogenous stem cell implantation strategy is finding suitable types of stem cells. Mesenchymal stem cells (MSCs) derived from various tissues are currently the most studied tissue engineering articular cartilage seed cell type [9]. Embryonic stem cells (ESCs) have the potential to differentiate into any cell type, but due to ethical disputes, ESCs are in only the preclinical experimental stage. Induced pluripotent stem cells (iPSCs) can theoretically be obtained by reprogramming any type of terminally differentiated cell, removing limitations of the cell source and reducing ethical disputes, thus becoming a new type of seed cell that is gradually emerging. However, stem cell transplantation also poses the risk of tumorigenesis, immune rejection, disease transmission, and the functional heterogeneity of cells from different individuals [10–13].

In this review, we first introduced the two main theories of stem cell-mediated articular cartilage regeneration and then reviewed the application of exogenous stem cell implantation strategies and endogenous stem cell homing and in situ cartilage regeneration strategies. Second, we reviewed the research progress of stem cell pretreatment strategies, derivatives, and delivery scaffolds. Finally, we summarized problems in stem cell research related to articular cartilage regeneration and looked toward the future directions of this field.

## 2. Theories on Cartilage Regeneration Involving Stem Cells

**Figure 1:**
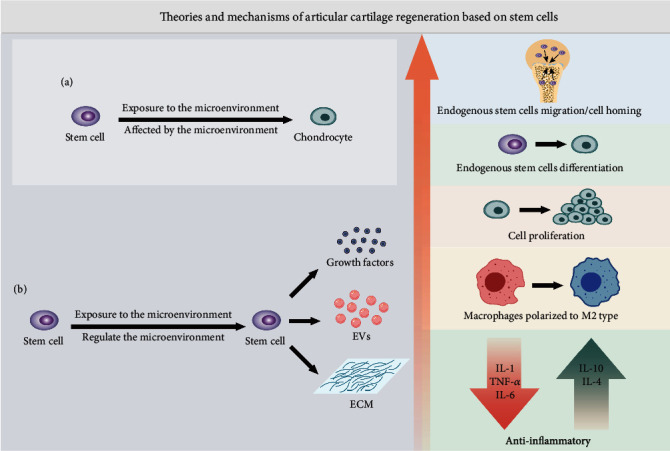
Two theories of articular cartilage regeneration involving stem cells. (a) Stem cell differentiation theory. Stem cells are affected by the microenvironment and directly differentiate into chondrocytes. (b) Paracrine theory of stem cells. Stem cells are affected by the microenvironment and secrete various derivatives, including growth factors, EVs, and ECM. These derivatives have been proven to induce homing of endogenous stem cells, promote the differentiation of endogenous stem cells into chondrocytes, promote the proliferation of chondrocytes, induce macrophages to polarize to the M2 type, and regulate the level of inflammatory factors to exert anti-inflammatory effects. EVs: extracellular vesicles; ECM: extracellular matrix.

As immature tissue precursor cells, stem cells can self-renew and have the ability to form clonal cell populations and differentiate into multiple cell lineages [14]. These special properties are particularly attractive for restoring the functions of a variety of organs. At present, stem cells can be divided into three general categories: (1) ESCs derived from early embryos, (2) iPSCs, and (3) adult stem cells, including hematopoietic stem cells, neural stem cells, and MSCs. A large number of studies have confirmed the beneficial role of stem cells in the regeneration of articular cartilage, and their potential mechanisms are mainly divided into two theories (Figure 1): the first is the “differentiation theory,” which states that stem cells directly differentiate into chondrocytes and repair damaged cartilage by adding or replacing chondrocytes [15]. The other is the “paracrine theory,” in which stem cells secrete bioactive factors, extracellular vesicles (EVs), and ECM [16], changing the biological behavior of receptor cells (including endogenous stem cells, chondrocytes, and macrophages), such as proliferation, differentiation, migration, polarization, metabolism, and apoptosis, and regulating the local microenvironment to repair and regenerate articular cartilage. Early studies focused on the direct differentiation and replacement of stem cells. In recent years, there has been an increasing amount of evidence that the therapeutic benefits of stem cells may be attributed to their paracrine effects.

### 2.1. Differentiation Theory

From the perspective of chondrogenesis, cartilage formation begins with mesenchymal condensation, which causes MSCs to differentiate into cartilage. Then, a dense matrix forms, which serves as a template for the subsequent formation of subchondral bone and cartilage [17]. In addition, a large number of studies have indicated that MSCs maintain pluripotency after repeated proliferation cycles *in vitro* and can differentiate into matrix-producing chondrocytes [18, 19]. Based on these findings, most previous studies attributed the role of stem cells in regenerating articular cartilage to their ability to differentiate into multiple lineages [20, 21]. A large number of studies focused on the development of materials and methods to induce stem cells to differentiate into cells with a chondrocyte phenotype [22]. Abir and colleagues demonstrated that autologous MSCs that were intra-articularly injected differentiated into mature chondrocyte-like cells [23]. This conclusion strongly supports this theory. Researchers suspended donkey autologous bone marrow-derived MSCs (BMSCs) labeled with green fluorescent protein (GFP) in hyaluronic acid (HA) for intra-articular injections in an attempt to treat wrist OA induced by amphotericin B. The results of up to 6 months of follow-up showed that the intra-articular injection of autologous BMSCs combined with HA resulted in an improved therapeutic effect compared with that of HA injections alone. GFP-labeled MSCs were detected in all the examined articular cartilage. Some cells showed a chondrocyte-like phenotype (round and surrounded by cavities), which proved that the injected MSCs differentiated into chondrocytes. To further verify this conclusion, similar work in a dog knee cartilage defect model also proved that injected MSCs differentiated into mature chondrocytes [24]. The same results were obtained in a study by Kotaka et al., who found that human iPSCs can repair knee cartilage defects in nude mice. The immunofluorescence of antihuman mitochondrial antibodies was found in newborn chondrocytes, which suggested that implanted iPSCs differentiated into chondrocytes [25]. In a recent study in a rat KOA model, researchers injected fluorescein-labeled human adipose-derived MSCs (ADSCs) into the articular cavity and found that the injected cells had a good therapeutic effect on OA. The existence of human cells in the rat meniscus and cartilage was confirmed by immunohistochemistry with antihuman mitochondria and antihuman Ki67 antibodies, and some of the cells were in the proliferative phase [26]. Although this study did not explore whether injected cells differentiated into mature chondrocytes, the fluorescence signal in OA rats lasted for approximately 10 weeks, which at least indicated that the implanted stem cells could be retained in the articular cavity for a long time. The above studies provide strong evidence for the “differentiation theory” of stem cells.

### 2.2. Paracrine Theory

Researchers have long known that the conditioned medium (CM) of stem cells can promote cell proliferation and differentiation *in vitro* and can promote tissue repair and regeneration *in vivo* [27]. It has been shown that stem cells secrete many cytokines and proteins. The synergistic effect of small molecules secreted by MSCs can reduce cell damage and improve the repair ability of tissue [28]. Second, the immunomodulatory effect of stem cells has been increasingly reported. Stem cells can regulate the immune microenvironment during the process of tissue repair and provide a good environment for tissue regeneration [29]. MSCs in the immune microenvironment can promote chondrogenesis through immune regulation [30]. At the same time, a large number of studies on the coculture of MSCs and chondrocytes *in vitro* have proven that paracrine signaling is an important feature of MSCs [31–33]. The nutritional function of MSCs has led researchers to increasingly regard them as therapeutic delivery agents, and it has been recommended to rename them “medicinal signaling cells” [34]. Their paracrine signaling drives the endogenous response [35]. On the other hand, some *in vitro* studies [36–38] found that the differentiation of MSCs is not as strong as originally thought, and it is difficult to achieve stable and effective differentiation. Especially in the case of differentiation into chondrocytes, the progression of stem cells to terminal hypertrophy is a frustrating problem [39]. Early *in vivo* follow-up studies showed that few cells can survive for more than a few weeks after implantation [40, 41]. A recent clinical study described the ultimate results of stem cell implantation. Tommy et al. implanted allogeneic MSCs into full-thickness femoral cartilage defects. After a 12-month repair period, histological samples were examined, and no allogeneic MSC DNA was detected in the repaired tissue. This indicated that implanted MSCs provided the initial stimulation but then died and were cleared from the tissue [42]. The above studies suggest that the function of stem cells in tissue repair and regeneration is mediated by active components secreted by stem cells rather than by their direct differentiation into target cells.

At present, the mechanism of stem cell-mediated cartilage regeneration is still unclear, and the above theory provides some insights. The complete regeneration process may be coordinated by multiple mechanisms, and stem cells may play different roles in different stages of the actual repair process. The precise control of the changing roles of stem cells may be an effective way to achieve the desired regeneration effect.

## 3. Cartilage Regeneration Strategies Involving Stem Cells

Stem cells used for tissue engineering and cell therapy are usually obtained from four basic sources: (1) embryonic tissue; (2) fetal tissue, such as fetus, amniotic fluid, and umbilical cord (Wharton jelly, blood); (3) a specific location in adult organisms (such as fat, bone marrow, and synovium); and (4) somatic cells after genetic reprogramming, i.e., iPSC [43, 44]. Among the sources of stem cells, adipose tissue seems to be the most promising choice. It have many unparalleled advantages. Specifically, adipose tissue is available in relatively high quantity in many patients and can be collected by “waste tissue” produced by surgical procedures (such as liposuction or abdominal plastic surgery), which can effectively solve problems with local morbidity, safety, and ethical issues. Moreover, compared with other tissues, adipose tissue produces a large number of living stem cells. Studies have shown that ADSCs in lipoaspiration account for 2% of nuclear cells, and the output per gram of adipose tissue is approximately 5000 fibroblast colony forming units (CFU-F). In contrast, the production of bone marrow MSCs (BMSCs) is only 100–1000 CFU-F/ml bone marrow [45]. Due to the tissue diversity and individual differences of MSC sources, the MSC population has obvious heterogeneity. Adult MSCs have obvious differences in their cartilage differentiation ability due to their different inherent tissue sources. Studies have compared adult MSCs derived from different tissues, and the results show that MSCs derived from joint synovium (SMSCs) have the strongest cartilage differentiation ability, which may be determined by their inherent cell characteristics and growth characteristics [46]. Researchers found high expression of proline arginine-rich end leucine-rich repeat protein (PRELP) in SMSCs, which is a glycoprotein rich in cartilage, but little or no content in stem cells outside the joints [47]. In addition, SMSCs remained multidirectional in 10 generations *in vitro*, and cell senescence was limited [48]. However, the acquisition of synovium is accompanied by invasive operation of the joint cavity, and the source of synovium is limited, which greatly limits the application of SMSCs. Compared with cells isolated from adult tissues, embryonic or neonatal-derived stem cells are characterized by faster proliferation and more passages *in vitro* before aging [49]. There is no study to compare the chondrogenic differentiation ability of neonatal/ESCs and adult stem cells, but studies have shown that single-cell-derived colonies of marrow stromal cells contained three morphologically distinct cell types: spindle-shaped cells, large flat cells, and very small round cells, and the small cells had a greater potential for multipotential differentiation [50].

With the development of high-throughput analysis technology, the heterogeneity of stem cells has become more obvious at the genetic molecular level [51]. Cell surface molecules that may be markers of stem cell pluripotency have been identified including but not limited to CD34 [52], CD146 [53], and CD49f [54]. Animal experiments show that CD146^+^ ADSCs can inhibit the inflammation of the joint cavity and promote the regeneration of articular cartilage [55]. Although no studies have confirmed the special role of CD34 and CD49f-specific stem cells in cartilage regeneration, the beneficial effects of CD34^+^ stem cells on cardiac repair and regeneration have been confirmed [56]. Studies have also found that the CM of CD34^+^ stem cells contains 32 soluble factors related to cell proliferation, survival, tissue repair, and wound healing, which can promote liver repair and regeneration *in vivo* [57]. MSCs with high expression of CD49f play an important role in the maintenance of hair follicle epithelial cells [58]. Directly implanting exogenous stem cells into joint cavities or articular cartilage defects seems to be the most direct stem cell application strategy. However, the strategy of stem cell homing and in situ regeneration was the first to be applied. Its history can even be traced back to 1959. Pridie [59] reported for the first time the subchondral bone drilling method used to treat cartilage injury. The bone marrow (containing BMSCs) was drained to the cartilage defect to form a blood clot, and then, cartilage tissue formed. However, there was no concept of “homing” stem cells at that time. This chapter will discuss these two strategies in detail.

### 3.1. Exogenous Stem Cell Implantation Strategy

**Figure 2:**
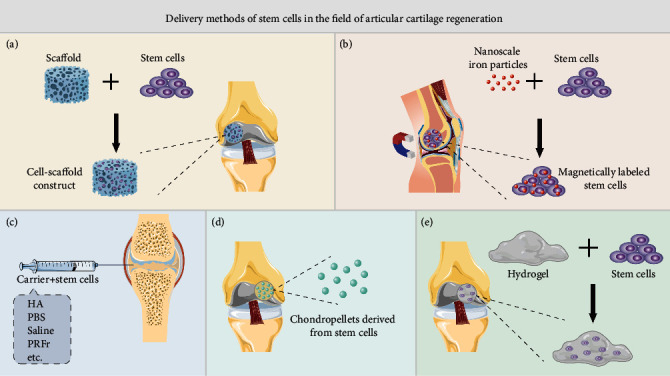
Stem cell delivery for repairing articular cartilage defects or treating OA. (a) Cell-scaffold construct. Stem cells are planted on a tissue engineering scaffold, cultured *in vitro* until the cells adhere to the scaffold, and then, the cell-scaffold construct is implanted into the cartilage defect. (b) Magnetic targeting. Place a magnet on the back of the cartilage defect (popliteal fossa), use nanoiron particles to label stem cells, and then implant the stem cells into the cartilage defect. Under the attraction of the magnet, the stem cells are tightly fixed to the bottom of the cartilage defect. (c) Intra-articular injection. The stem cells are resuspended in hyaluronic acid (HA), phosphate-buffered saline (PBS), physiological saline or platelet-rich fibrin releasate (PRFr), and other carriers and then injected into the joint cavity. (d) Chondrocyte pellets. The stem cells are cultured and differentiated *in vitro* to form cartilage pellets, and then, the cartilage pellets are implanted into the cartilage defect. (e) Cell-hydrogel construct. The stem cells are mixed into the injectable hydrogel material, and then, the cell-hydrogel construct is injected into the cartilage defect.

We searched for studies applying exogenous stem cell implantation strategies to treat articular cartilage defects or OA on PubMed from the past 4 years (2017-2020) and summarized the representative studies in Table 1 (animal experiments) and Table 2 (clinical research). According to the search results, most studies showed good therapeutic effects. Most of the animal models used in animal experiments involved rats, rabbits, pigs, sheep, and horses. The pathological process of OA in these quadrupeds may be quite different from that in humans. One study used a model of OA in primates (rhesus monkeys) [60]. Encouragingly, the results of this study showed that both xenogenic ESC-derived MSCs (EMSCs) and allogeneic BMSC transplantation had therapeutic effects on knee joint OA in rhesus monkeys, and the results were better than those in the control group. There are relatively few clinical studies, and there are only 2 clinical studies with a large sample (more than 100 cases) [61, 62]. In terms of the follow-up time, the evaluation time for animal experiments ranged from 3 weeks to 64 weeks. The shortest follow-up time for a clinical study was 6 months, and the longest follow-up time was more than 36 months. Because articular cartilage is in an ischemic and hypoxic environment that relies on only synovial fluid to supply nutrients, the regeneration of articular cartilage often takes a long time [63]. Therefore, long-term follow-up has more reference value. In terms of the stem cell dose, the single dose used in most studies was 10^6^-10^7^ cells. Although a higher number of cells would theoretically increase the number of successful stem cell transplants, there may be a plateau, beyond which the results will not continue to improve. For example, a study by Wu et al. confirmed that the intravenous injection of 1 × 10^6^ MSCs improved the neurological function of rats with brain injury, but increasing the dose to 3 × 10^6^ cells did not lead to a greater improvement in function [64]. In addition, some studies have shown that the repeated delivery of stem cells can have a better therapeutic effect [65], and no serious adverse events, such as tumorigenesis, were found during the 2-year follow-up. However, increased treatment costs, tedious cell culture and expansion procedures, and potential infection risks are problems that cannot be ignored. The delivery mode of stem cells determines the success rate of stem cell transplantation to some extent. We summarize the commonly used delivery methods in Figure 2. Because of the lack of blood vessels in articular cartilage, it is difficult to deliver drugs through the intravenous or arterial system. Most studies directly inject stem cells into the articular cavity, usually using normal saline, phosphate-buffered saline (PBS), or HA as cell carriers. After the direct injection of stem cells into the articular cavity, it is impossible to accurately target the area of cartilage injury. Although studies by Xia et al. [66] and others have shown that superparamagnetic iron oxide-labeled BMSCs gather at the location of cartilage defects after injection into the articular cavity, the practicability of the technique needs to be further verified. Magnetic targeted delivery and cell-scaffold constructs may solve this problem, but magnetic targeted delivery is still in the preclinical research stage [25], and the long-term effect of magnetic iron particles on cell and tissue regeneration is unclear. The cell-scaffold construct strategy has been used in the clinic. According to the search results, three commercial scaffold products have been used [62, 67, 68]. This may be due to the incomplete supervision and management policies of various countries on cell products, especially stem cell products, which restricts the translation of related products into clinical practice. Although there are still few commercial products of stem cell-scaffold constructs at present, commercial products of autologous chondrocyte-scaffold constructs have been widely used, and their therapeutic effects are ideal [69]. We have reason to believe that stem cells with stronger proliferation and differentiation ability have better application prospects.

A large amount of clinical follow-up evidence has proven that MF, cartilage transplantation, ACI, etc., can regenerate fibrocartilage, but the long-term treatment effects are not good. An increasing number of scholars have attempted to combine stem cell transplantation with these traditional repair methods. Song et al. [62] combined human umbilical cord blood-derived MSC transplantation with MF, and Kim et al. [67] combined autologous ADSC transplantation with allogeneic cartilage transplantation (MegaCartilage, particulate allogenic cartilage, L&CBio, Seoul, KR), which significantly improved the clinical symptoms of OA patients. These results provide a reference for the combined use of stem cells and traditional cartilage repair techniques.

However, the limitations of exogenous stem cell implantation strategies cannot be ignored, such as the risk of tumorigenesis, the risk of disease transmission, the risk of immune rejection, and the restrictions on stem cell regulatory policies in different countries.

### 3.2. Stem Cell Homing and In Situ Regeneration Strategy

The term “homing” was first proposed by Gallatin et al. [92] in 1983. It was first used to describe the phenomenon in which lymphocytes in circulating blood tend to migrate to the sites that they were originally derived from, such as lymph nodes, which is referred to as “lymphocyte homing,” and then was gradually extended to stem cells. The term has recently been used to emphasize the ability of stem cells to respond to extracellular signals, such as migration stimuli and guidance cues, for targeted transport and migration [93]. Most tissues initiate the recruitment of stem cells to a certain extent when they are injured or inflamed, which promotes the homing of stem cells to the damaged area and exerts the potential for a variety of repair types, including ECM reconstruction and microenvironment regulation [94, 95]. Recruited stem cells can come directly from the stem cell pool of the tissue around the injury or be recruited from the circulatory system. As endogenous stem cells/progenitor cells do not need to be cultured and expanded *in vitro* and there is no risk of immunogenicity and disease transmission, researchers have focused on in situ cartilage regeneration by triggering endogenous stem cells/progenitor cells to undergo “homing” [96].

To enhance the homing behavior of stem cells, researchers tested the following strategies.

#### 3.2.1. Artificially Increasing the Concentration of Chemokines in the Injured Site

For example, the stromal cell-derived factor (SDF-1)/CXCR4 signaling pathway has been shown to play a key role in endogenous stem cell homing [97, 98]. Zhang et al. successfully repaired part of a thickness cartilage defect in a rabbit knee joint with a type I collagen scaffold containing SDF-1 and confirmed that increasing the concentration of chemokines at the injured site promoted the homing of endogenous stem cells and mediated cartilage regeneration [99]. In another recent study, researchers embedded transforming growth factor *β*1 (TGF-*β*1) in photocrosslinked glycidyl methacrylate (GM-HPCH) to repair articular cartilage defects in rats. The results showed that compared with GM-HPCH alone, GM-HPCH+TGF-*β*1 could repair cartilage defects more effectively through its ability to recruit stem cells [100]. In similar studies, increases in interleukin 8 (IL-8) and macrophage inflammatory protein 3*α* (MIP-3*α*) were shown to promote stem cell homing to articular cartilage injury sites and mediate articular cartilage regeneration [101].

#### 3.2.2. Increasing the Number of Stem Cells in the Damaged Local Microenvironment

For example, MF can stimulate and release BMSCs. Min and others demonstrated that the MF channel caused by the hollow cone is more unobstructed than that caused by a traditional blunt cone and can mobilize more BMSCs to the location of a cartilage defect [102]. Baboolal et al. stirred joint synovium with a special stem cell mobilization device (StemDevice) for 1 minute and collected joint cavity lavage fluid for cell culture. Compared with ordinary cytological brushes, this stem cell mobilization device greatly increased the number of synovial stem cells in the lavage fluid [103]. Encouragingly, both of these techniques have been applied in the clinic, and both are arthroscopic-assisted operations with the advantages of being minimally invasive.

#### 3.2.3. Construct Scaffolds Conducive to Stem Cell Homing, Adhesion, Proliferation, and Differentiation

For example, Sun et al. combined self-assembled peptide nanofiber hydrogels (RAD/SKP) with acellular cartilage matrix (DCM) scaffolds. It was confirmed in animal experiments that the DCM-RAD/SKP functional scaffold system significantly promoted the recruitment of endogenous stem cells and regenerated hyaline cartilage [104].

It is worth noting that at present, many studies are not limited to the application of one of these strategies, but a variety of strategies can be combined to improve the repair effect. In a recent study, researchers first used 3D-bioprinting technology to construct a silk fibroin-gelatin composite scaffold (SFG), which had a porous structure suitable for cell adhesion and good mechanical strength. The scaffold was then combined with a BMSC-specific affinity peptide (E7), which was shown to have the ability to recruit BMSCs. In the rabbit knee articular cartilage defect model, the SFG-E7 composite scaffold was combined with MF. After 24 weeks, the cartilage defect was completely filled, and the new tissue had obvious characteristics of hyaline cartilage [105]. The research team modified the acellular porcine peritoneal matrix (APM) scaffold with the E7 polypeptide, which had good biocompatibility and a surface suitable for cell growth. The combined application of the APM-E7 scaffold and MF greatly enhanced the recruitment of endogenous stem cells and regenerated rabbit knee cartilage [106].

Endogenous stem cell recruitment and in situ regeneration strategies also face many limitations. The biologically active ingredients used to recruit stem cells often require high synthesis techniques and conditions. At the same time, in order to exert a sustained recruitment effect, the delivery materials need to have a slow-release function.

## 4. Stem Cell Pretreatment Strategy

The microenvironment of damaged articular cartilage is adverse, with inflammation, hypoxia, and insufficient blood supply. In addition, most stem cells used in clinical applications come from adults, and the functions of these cells are compromised. The above factors lead to a very low survival rate of transplanted cells [107], and the use of stem cells for cartilage regeneration has not yet achieved the desired effect. Studies have shown that pretreatment is an effective way to enhance the ability of stem cells to resist adverse microenvironments. Stem cell pretreatment can improve cell survival and differentiation potential, regulate the immune response, inhibit fibrosis, and enhance cell secretion of anti-inflammatory factors. These effects promote the regeneration and functional recovery of organs and tissues after cell implantation [108, 109]. Stem cell pretreatment strategies reported in the field of cartilage regeneration mainly include the following aspects:

### 4.1. Hypoxia

In natural cartilage, cells are exposed to very low oxygen pressure: approximately 7% (53 mmHg) in the superficial area and only 1% (5-8 mmHg) in the deep area [110]. Hypoxic pretreatment not only enhances the survival and migration ability of stem cells after implantation but also promotes the proliferation and differentiation of stem cells [111]. Under the same conditions for cartilage-induced differentiation, compared with MSCs without hypoxia pretreatment, MSCs with hypoxia pretreatment have been shown to enhance matrix deposition and reduce the expression of hypertrophy markers such as type X collagen [112]. Additionally, hypoxic pretreatment can also upregulate genes related to growth, cell signaling, metabolism, and cellular stress response pathways [113]. In a rabbit knee joint trauma and focal early OA model, hypoxia-pretreated MSC+HA hydrogel caused a significant improvement in the cartilage repair score [114]. The mechanism through which hypoxia affects cells is mainly regulated by HIF-1. The latest evidence shows that HIF-1*α* promotes cartilage matrix gene expression and upregulation and that HIF-3*α* can help stabilize the cartilage phenotype. In contrast, HIF-2*α* upregulates hypertrophy genes and matrix-degrading enzymes [112]. Some studies have explored the specific mechanism of hypoxia that regulates HIF. Studies have shown that hypoxia can induce an increase in phosphorylated AKT and p38 MAPK to stabilize HIF-1*α* [115], resulting in the upregulation of the glucose-6-phosphate transporter and an increase in the MSC survival rate [116].

### 4.2. Pharmacological or Chemical Agents

The use of pharmacological or chemical reagents to protect stem cells and improve the effect of stem cells on cartilage regeneration is another pretreatment strategy. For example, vitamin E pretreatment can make MSCs resistant to H_2_O_2_-induced oxidative stress, upregulate the expression of proliferation markers and transforming growth factor-*β* (TGF-*β*), and downregulate the expression of apoptosis-related genes. After the above pretreatment, MSCs increased the content of proteoglycan in the cartilage matrix in a surgically induced OA rat model, upregulated a chondrogenesis marker, and promoted the differentiation of MSCs into cartilage [117]. Kartogenin (KGN) has been proven to be a chondrogenesis and cartilage protective agent that is more effective in inducing cartilage regeneration than growth factors [118]. Jing and colleagues found that KGN pretreatment may improve the chondrogenesis and differentiation of human WJMSCs by promoting human WJMSCs to enter the prechondral phase, enhancing JNK phosphorylation and inhibiting dicatenin [119]. A recent study found that EVs derived from human WJMSCs pretreated with KGN contain a unique miRNA, miR-381-3p. Researchers found that miR-381-3p directly inhibited TAOK1 by targeting the 3′ untranslated region of TAOK1, thus inhibiting the Hippo signaling pathway and mediating cartilage formation [120].

### 4.3. Trophic Factors and Cytokines

The interaction between specific nutritional factors and their receptors can activate downstream signal transduction and promote cell survival and differentiation. Therefore, the pretreatment of stem cells with nutritional factors and cytokines is a promising strategy for improving the therapeutic effect of stem cells. Stem cells pretreated with FGF-2 have been shown to have an enhanced proliferation ability and to retain the potential to differentiate into cartilage after 30 population doublings, while stem cells that were not pretreated lost their ability to differentiate into cartilage after approximately 20 doublings [121]. The pretreatment of stem cells with specific growth factors can promote their chondrogenic differentiation potential and their ability to repair cartilage defects *in vivo* [111]. For example, pretreatment with an appropriate concentration of IL-1*β* can not only promote proliferation but also enhance the chondrogenic potential of synovial MSCs. However, high concentrations of IL-1*β* adversely affected synovial MSCs by reducing their adhesion and pluripotency [122]. BMSCs pretreated with soluble IL-6R effectively repaired articular cartilage defects *in vivo* [123].

### 4.4. Physical Factors

Articular cartilage is a load-bearing tissue, so mechanical stimulation is very important for the development and maintenance of articular cartilage. A 3D culture model can mimic the natural growth state of cells *in vivo*, provide enough space for stem cell proliferation, and produce more biochemical and biomechanical clues by providing intensive cell-to-cell interactions. Therefore, with these advantages, a variety of physical factors can be applied to the 3D microenvironment *in vitro* or *in vivo* to improve the performance of stem cells [124]. For example, Zhang et al. found that radial shock waves not only significantly improved the proliferation and self-renewal ability of MSCs *in vitro* but also safely promoted the repair cartilage defects by MSCs *in vivo* [125]. The articular cartilage matrix contains a large amount of collagen type II (COLII), and the expression of the SOX9 gene is positively correlated with COLII. Continuous low-intensity ultrasound pretreatment upregulated SOX9 gene expression and enhanced the nuclear localization of SOX9 protein in MSCs compared with control stimulation by discontinuous low-intensity ultrasound [126]. In addition, a new method involves combining cells with carriers/scaffolds before physical stimulation. To date, researchers have designed different types of cell carriers with appropriate physical and chemical properties for cell transplantation, such as injectable hydrogels, large scaffolds, microcarriers, and microspheres [127]. Cheng et al. loaded BMSCs onto an autologous platelet-rich fibrin (PRF) membrane scaffold and applied hydrostatic pressure to the cell-scaffold construct before transplantation. The results showed that the cell scaffold pretreated by hydrostatic pressure significantly increased the formation rate and matrix content of new cartilage and enhanced its mechanical properties [128].

### 4.5. Genetic Modification

A large number of studies have genetically engineered stem cells to reduce their tendency to differentiate into a hypertrophic phenotype or to induce the overexpression of transcription factors and growth factors to promote the formation of new cartilage *in vivo* [129, 130]. The overexpression of specific growth factors before implantation is a controllable and effective way to improve the efficacy of stem cell therapy. Genes for specific factors can be introduced into cells by nonviral or viral techniques. For example, compared with simple cellular or acellular scaffolds, BMSCs overexpressing TGF-*β*1 can be implanted into polylactic acid (PLA) scaffolds to achieve good cartilage tissue repair in a rabbit knee osteochondral defect model [131]. With regard to the specific progress of gene modification in cartilage repair, please refer to relevant reviews [129, 130].

There are still few *in vivo* animal experiments on stem cell pretreatment strategies, and there is currently a lack of standard protocols. The optimal length and dosage of stem cells need to be explored in depth. At the same time, it is necessary to clarify the molecular mechanism of physical, chemical, and genetic processing methods to promote cartilage regeneration.

## 5. Composition and Characteristics of Stem Cell Derivatives for Cartilage Regeneration

Stem cell derivatives are an extension of the paracrine theory of stem cells (Figure 1), in which the secretome is considered to be the mechanism through which stem cells exert their tissue repair and regeneration effects [132]. The secretome is a general term for bioactive factors and EVs secreted from the cell to the extracellular space. The secretome of cells is specific but changes in physiological or pathological conditions that directly affect it [133]. Bioactive factors include growth factors, cytokines, chemokines, and enzymes [134]. EVs are considered an important component of the therapeutic efficacy of MSCs. According to the size, composition, and origin of EVs, they can be divided into three types: apoptotic bodies, microvesicles, and exosomes [135, 136]. There are relatively few studies on apoptotic bodies, which are generated only during apoptosis, have a diameter of 50-5000 nm, and carry nuclear fragments and organelles. Microvesicles are small vesicles with a diameter of 50-1000 nm released by cells in the form of budding, which can be obtained by 10,000-20,000 g centrifugation. Exosomes are formed by the multivesicular endosomal pathway and are usually a complex of proteins, nucleic acids, and lipids with a diameter of 50-200 nm that can be obtained from very high-speed centrifugation at or above 100,000 g. Although stem cells have become powerful tools for clinical applications, they still have limitations in terms of delivery, safety, and the heterogeneity of therapeutic responses. The secretome composed of cytokines, chemokines, growth factors, proteins, and EVs may represent an effective alternative [16]. Notably, MSC-derived EVs (MSC-EVs) have been demonstrated to have a similar effect to MSCs and may have advantages over parent cells because of their specific miRNA load [135]. The focus of current research has shifted from stem cells to their secretome while attempting to overcome the limitations of cell-based therapies.

In addition, stem cell-derived ECM can be obtained by decellularizing stem cells cultured *in vitro*, and the ECM is a noncellular component that contains macromolecules secreted by various cells. The ECM may vary among cell type sources, but it is mainly composed of proteoglycans, such as growth factors, glycosaminoglycan (GAG), and matrix proteins, as well as collagen, fibronectin, elastin, vitronectin, and laminin [137]. After removing cellular components, such as DNA and cellular components that trigger an immune response, the ECM retains natural biochemical and biophysical signals [138]. Recent studies have shown that the ECM can promote cell proliferation and chondrogenic potential and is a potential biomaterial for tissue-engineered articular cartilage [139, 140].

The following sections will discuss in detail three aspects of the application of stem cell derivatives in cartilage regeneration and OA treatment: stem cell-derived CM, purified EVs (microvesicles and exosomes), and stem cell-derived ECM.

### 5.1. Stem Cell-Derived CM

Compared with stem cells, CM can be stored in a low-temperature environment, which is convenient for transportation, and does not have the risk of tumorigenesis. Compared with EVs and certain growth factors, CM components are more complex, including all components of the cell secretome, and do not need to be isolated and extracted, making it is convenient to use. Recently, Islam et al. studied the secretome of stromal cells obtained from the Hoffa fat pad (HFPSCs), synovial (SMSCs), umbilical cord (UCSCs), and cartilage (ACs) by quantitative liquid chromatography-mass spectrometry (LC-MS/MS) proteomics [141]. They identified more than 1000 proteins in each type of cell-derived CM. The secretome contained a large number of growth factors and cytokines. More importantly, compared with stromal cells from adult tissues, UCSCs had stronger anti-inflammatory and immunosuppressive properties. Recent studies reported that stem cell-derived CM plays a role in anti-inflammation and immune regulation and increases the synthesis of cartilage matrix in arthritis and osteochondral defect models. Ishikawa et al. intravenously injected CM derived from human dental pulp stem cells into the joint cavity of rheumatoid arthritis mice and found that CM relieved joint symptoms and synovial inflammation. The histological scores of bone erosion and cartilage damage in the CM group were significantly better than those in the control group, and the gene expression levels of proinflammatory cytokines were significantly reduced [142]. Alasdair found that the intra-articular injection of MSC-CM reduced cartilage damage and inhibited the immune response by reducing the cleavage of aggrecan, enhancing Treg function, and regulating the ratio of Treg : Th17 [143]. In addition, the application of BMSC-CM in a rat model of antigen-induced arthritis significantly reduced edema and thermal hyperalgesia as well as serum levels of TNF-*α* [144]. The anti-inflammatory effect of CM is related to its various immunomodulatory factors, including TGF-*β*, thrombospondin 1 (TSP1), and prostaglandin E2 (PGE2) [134]. Moreover, MSC-CM can also inhibit the progression of OA by balancing the ratio of MMP-13 to TIMP-1 in cartilage, inhibiting chondrocyte apoptosis and enhancing autophagy [145]. In a rabbit osteochondral defect repair experiment, the application of BMSC-CM led to only fibrocartilage regeneration [146]. Widhiyanto et al. composited ADSC-CM into porous scaffolds to repair rabbit trochlear cartilage defects, and new hyaline cartilage was observed at 12 weeks [147]. Interestingly, contradictory results were reported in a rabbit ear cartilage regeneration study. Researchers subcutaneously injected ADSCs, ADSC-CM, and PBS and found that there was no significant difference between the ADSC-CM and PBS groups at 4 or 8 weeks [148]. The above studies preliminarily proved that stem cell-derived CM repaired articular cartilage defects and relieved OA. The differences in experimental results *in vivo* may be related to the application method. When using scaffolds as carriers, CM can be retained in the defect area and gradually released. Stem cell-derived CM contains the whole secretome, and different stem cells and pretreatments can significantly affect the composition of CM. Researchers need to find more effective CM collection conditions to promote cartilage regeneration and to ensure that there are effective concentrations of effector substances at the target location to achieve better cartilage regeneration. Researchers also need to determine the exact biological mechanism of CM *in vivo*.

### 5.2. Stem Cell-Derived EVs

Unlike the direct use of stem cell-derived CM, EVs need to be separated and purified. The current methods used to obtain EVs include but are not limited to ultrafiltration and size-exclusion chromatography [149, 150], ultracentrifugation [151], and immunoaffinity [152]. Recently, an increasing number of reports have indicated that exosomes are the main therapeutic agents secreted by MSCs that enhance the regeneration and immunomodulatory ability of MSCs during tissue repair [135]. It has been reported that stem cell-derived EVs can promote cartilage regeneration and prevent cartilage degeneration induced by OA [153–156]. In a rat model of osteochondral defects, the weekly injection of human ESC-derived exosomes into the joint cavity induced cartilage and subchondral bone tissue regeneration within 2 weeks, and the orderly regeneration of the two tissues was observed at 12 weeks [153]. Compared with MSC injection, a single intra-articular injection of exosomes or microvesicles derived from mouse BMSCs had similar effects in preventing the development of collagenase-induced OA in mice [154]. Exosomes derived from human ESCs also showed cartilage protective effects in a mouse OA model [155]. Another study compared the therapeutic effects of iPSC-derived exosomes and synovial-derived exosomes in a collagenase-induced mouse OA model. The results showed that iPSC-derived exosomes could more effectively delay the progression of OA [157]. The biodistribution of EVs after intra-articular injection is not clear. Encapsulating EVs in a suitable biomaterial can prevent the rapid clearance of EVs and achieve a sustained release effect. Liu et al. encapsulated hiPSC-MSC-derived exosomes in a photocrosslinked hydrogel, which resulted in the retention of exosomes *in vitro* and achieved cartilage regeneration and repair in a rabbit femoral condylar cartilage defect model [63]. Chen et al. used desktop stereolithography to fabricate 3D-printed cartilage ECM/methacrylic acid gelatin (GelMA)/exosome scaffolds with radial channels. *In vivo* experiments showed that the 3D-printed scaffolds significantly promoted cartilage regeneration [158]. *In vitro* mechanistic studies showed that EVs derived from MSCs mediate cartilage repair by enhancing proliferation, reducing cell apoptosis, and regulating the immune response [159].

With the in-depth study of the therapeutic mechanism of EVs, the anti-inflammatory effects of EVs have been reviewed in detail [160, 161]. A growing number of scholars believe that the therapeutic efficacy of EVs can be attributed to their nucleic acid composition [162]. An increasing number of studies have described a complex picture of how miRNA regulates or influences OA [163]. Wu et al. reported that ADSC-derived exosomes from the human subpatellar fat pad protected articular cartilage from damage and improved gait abnormalities in OA mice by maintaining cartilage homeostasis, which may have been related to the inhibition of the mTOR autophagy pathway regulated by miR100-5p [156]. Another study proved that exosomes derived from SMSCs with high miR-140 expression promoted articular cartilage regeneration in rats [164]. In addition, early molecular mechanism studies showed that miR-92a regulates the PI3K/AKT/mTOR signaling pathway by targeting noggin3, thus upregulating chondrocyte proliferation and matrix synthesis [165, 166]. Exosome miR-23b induced human MSCs to differentiate into chondrocytes by inhibiting the protein kinase A (PKA) signaling pathway [167]. On the other hand, miR-125b and miR-320 reduced ECM damage by downregulating the expression of aggrecanase-1 (ADAMTS-4) and MMP-13, while these two ECM proteases were significantly upregulated in human OA chondrocytes [168]. Recently, Enrico et al. conducted high-throughput screening of the human adipose-derived MSC secretome and identified 60 kinds of miRNAs that can protect cartilage and induce macrophages to polarize to an M2 phenotype through bioinformatics analysis [169]. Increasing evidence indicates that stem cell-derived EVs may promote cartilage regeneration and treat OA by regulating a complex miRNA network [163]. Finally, the application of stem cell-derived EVs in treatment may have more advantages than using stem cells alone, mainly for the following reasons: (1) they cannot proliferate and are easy to preserve and transport [170]; (2) EVs are nontoxic, have no risk of tumorigenesis, low immunogenicity, and higher safety [171]; and (3) compared with the regulatory and ethical restrictions on stem cell products, the application of EVs is less restricted. However, in the field of cartilage repair, there are still many questions about the therapeutic effect, biodynamics, biodistribution, and delivery methods of stem cell-derived EVs that need to be answered in large animal experiments.

### 5.3. Stem Cell-Derived ECM

Stem cell-derived ECM is a natural biomaterial with strong biological activity and good biocompatibility. A large number of studies have shown that stem cell-derived ECM can enhance cell proliferation, prevent chondrocyte dedifferentiation, and maintain the stemness of stem cells [172, 173]. Stem cell-derived ECM provides a better platform for the expansion of chondrocytes/stem cells *in vitro*. Many studies have shown that compared with tissue culture polystyrene (TCPS), stem cell-derived ECM can significantly improve the proliferation of chondrogenic cells. At the same time, chondrogenic cells expanded on stem cell-derived ECM have stronger chondrogenic potential [174, 175]. Pei et al. showed that compared with cell culture plates, porcine synovial stem cell-derived ECM increased the proliferation of chondrocytes and delayed the dedifferentiation of porcine chondrocytes [174]. At the same time, stem cell-derived ECM can be used as a substrate for stem cell culture *in vitro*, which can restore the lineage differentiation ability of stem cells in aging mice [176]. Research by Yang et al. showed that compared with chondrocytes grown on TCP, chondrocytes inoculated on human BMSC-ECM showed a significantly increased proliferation rate and maintained a better cartilage phenotype. After expanding to the same number of cells and placing them in high-density micromass culture, chondrocytes from the BMSC-ECM group showed better cartilage differentiation characteristics than those from the TCP group [175]. Interestingly, the age of host that cells were derived from and different cell sources seem to be important factors affecting the ECM. Chee et al. found that fetal BMSC-ECM was superior to adult BMSC-ECM or human neonatal dermal fibroblasts in promoting the proliferation and pluripotency of adult BMSCs [177]. In addition to promoting cell proliferation and lineage-specific differentiation, recent studies have shown that SMSC-ECM enhanced the anti-inflammatory properties of rabbit articular chondrocytes through the SIRT1 pathway [178].

In addition to being used as a cell culture substrate, stem cell ECM can also be used alone or in combination with polymer materials to make 3D scaffolds to promote cartilage formation *in vivo*/*in vitro*. Tang et al. evaluated the cartilage repair ability of autologous BMSC-derived ECM scaffolds in two kinds of cartilage defect animal models. Six months after surgery, the histological characteristics and biochemical content of the bone marrow stimulation + ECM group were similar to those of normal hyaline cartilage [179]. Makiko et al. inoculated human amniotic MSCs on PLGA, successfully prepared an ECM-PLGA scaffold by removing cellular components, and implanted the scaffold into an osteochondral defect in the rat femoral trochlea. The results showed that ECM-PLGA induced gradual tissue regeneration and resulted in hyaline cartilage repair that was superior to that in the empty control group [180].

Current research shows that various stem cell derivatives play beneficial roles in cartilage regeneration and OA treatment. However, compared with the direct application of stem cells, the most substantial problem faced by stem cell derivatives is the cumbersome collection process, which undoubtedly increases the cost of treatment. In addition, how to increase the yield of exosomes and other derivatives and ensure the unity between batches is an urgent problem to be solved.

## 6. Stem Cell Delivery Biomaterials and Scaffolds

The key factor determining the effectiveness of stem cell therapy is the survival rate of stem cells during and after transplantation. Biomaterials used for cartilage repair not only provide mechanical support for cartilage defects but also provide support matrix for stem cells to induce cell growth, diffusion, and differentiation [181]. Biomaterial-based cell delivery systems can be extracted from naturally occurring materials, such as HA [182], gelatin [183], alginate [184], collagen, and decellularized matrix [185, 186] or based on synthetic materials, such as poly(ethylene glycol) (PEG) [187], poly(N-isopropylacrylamide) (PNIPAM) [188], poly(lactic-co-glycolic acid) (PLGA) [189], and polycaprolactone (PCL) [190]. The material is usually made into a porous structure to facilitate cell inoculation or hydrated polymeric networks, hydrogels for cell encapsulation [191]. Natural materials have better biological effects such as promoting cell adhesion, proliferation, and cartilage differentiation [192]. However, the mechanical properties and degradation rate of synthetic materials are more adjustable, and it is easier to customize according to cartilage or bone cartilage [193]. By combining biomaterials or natural ECM components with synthetic polymers, it is beneficial to highlight their respective advantages while limiting their disadvantages [194–197].

Early researchers used the material as a stem cell delivery platform to ensure the survival rate of stem cell transplantation to the defect and enhance the cell retention and therapeutic effect at the local administration site. Vahedi et al. inoculated adipose-derived stem cells into PCL scaffolds, and the ASC-PCL construct treated with low-intensity ultrasound achieved effective cartilage regeneration in a sheep model of a femoral condylar cartilage defect [198]. Collagen exists widely in a variety of biological tissues, has good biocompatibility and biodegradability, and has good plasticity [199]. Shi et al. successfully fabricated silk-fibroin-gelatin composite scaffolds using 3D printing technology and introduced BMSC-specific-affinity peptide [105]. This composite scaffold not only provides a suitable three-dimensional structure for stem cell proliferation, differentiation, and extracellular matrix synthesis but also achieves articular cartilage regeneration by recruiting endogenous BMSCs. In cartilage tissue engineering, the use of decellularized ECM is a relatively new concept. Our study group has proven that decellularized cartilage ECM porous scaffolds can promote stem cell adhesion, proliferation, and cartilage differentiation. At the same time, preclinical studies have proven that decellularized cartilage scaffolds have an excellent cartilage repair effect [200–203].

Although collagen type II and HA are key components of cartilage ECM, mainly type I collagen and HA have been developed as hydrogels for experimental and clinical cartilage repair [204]. To develop injectable scaffolds for the treatment of cartilage, the effects of HA hydrogel on chondrogenic differentiation and cartilage repair of hMSCs have been evaluated *in vitro* and *in vivo*. Result showed that the hydrogels reduce the fast leakage of the encapsulated growth factors, leading to the enhanced chondrogenesis of hMSCs and neocartilage formation [205]. Hydrogel can also achieve better cartilage repair by encapsulating functional biological small molecules. Xu et al. demonstrated that hydrogel encapsulation resulted in more sustained release of kartogenin and TGF-*β*1, which led to enhanced chondrogenesis of encapsulated human bone marrow MSCs *in vitro* and *in vivo* [206]. For the treatment of cartilage and osteochondral defects, the exact size and shape can be determined only after debridement. Therefore, methods such as in situ 3D bioprinting or hydrogel application are the most appropriate procedures for providing personalized treatment. The customizability of traditional solid scaffolds is weak, while the limitations of hydrogels include poor mechanical integrity and rapid degradation when exposed to inflammatory environment [207]. With the deepening of the intersection of biology and material manufacturing disciplines, any strategy aimed at imitating the composition and regional organization of articular cartilage will be more likely to reconstruct engineering tissue with the potential for successful clinical application [208].

The current biomaterials and scaffolds used for the delivery of stem cells still have many problems to be solved. For example, the biocompatibility of polymer materials is poor, and their degradation products may cause changes in the pH of the microenvironment. The mechanical properties and degradation rate of natural biomaterials are difficult to control, and its activity and functionality in the body are still to be clarified.

## 7. Conclusions and Future Perspectives

In the field of articular cartilage regeneration and OA treatment, research involving stem cells has moved from the laboratory to the clinic [209, 210]. However, several problems remain that restrict the application of tissue-engineered cartilage involving stem cells.

First, the functional heterogeneity of stem cells is a substantial obstacle to their clinical transformation [211]. Therefore, before using stem cells, it is necessary to screen out specific subgroups to more accurately explore the molecular mechanism of cartilage regeneration. Second, the problem of premature differentiation of stem cells *in vitro* expansion has not been resolved. Finding specific targets that regulate stem cell differentiation may solve this problem. For example, methyltransferase inhibitors can inhibit Setd7 protein, prevent cell differentiation, and maintain cell division. Researchers used stem cells containing methyltransferase inhibitors to treat muscle atrophy mice, and the results showed that the strength of regenerating muscle was significantly improved [212]. Finally, standard animal models of articular cartilage defects and OA have not yet been established [213]. Rodents such as mice and rats maintain open endochondral ossification throughout their lives, and the healing of cartilage defects may be greatly affected by spontaneous internal healing [214]. Using large animals such as pigs and horses often limits the number of samples due to high prices. Therefore, finding a balance between effectiveness and economic benefits is necessary in the choice of animal models.

The use of stem cell derivatives to regenerate articular cartilage is a promising development direction [133]. miRNA is considered to be the main component that mediates the biological effects of EVs. However, the main problem currently encountered is that it is technically challenging to produce a sufficient number of EVs, and the amount of nucleic acid packages for EVs is too low [215, 216]. Cell nanoporation biochips can not only increase the production of exosomes but also realize the encapsulation of specific miRNAs [217]. This new technology may help translate the EV-based cartilage regeneration strategy into clinical practice.

**Figure 3:**
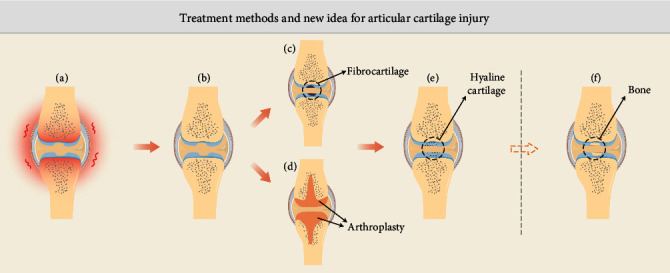
Treatment methods and new idea for articular cartilage injury. (a) Joint cartilage defects cause joint inflammation. (b) Medication can relieve symptoms. (c) Traditional repair techniques such as MF form fibrocartilage. (d) Artificial joint replacement surgery reconstructs the articular surface. (e) Ideal form of cartilage regeneration. (f) New ideas for cartilage regeneration.

The treatment of articular cartilage defects has gone through several stages of development: drug treatment can only relieve symptoms (Figures 3(a) and 3(b)). MF and other techniques often use fibrocartilage to temporarily fill cartilage defects (Figures 3(b) and 3(c)) [218]. Artificial joint replacement surgery temporarily restores the smooth joint surface, but the artificial material has a limited life span (Figures 3(b) and 3(d)). The use of hyaline cartilage to restore the joint surface (Figure 3(e)) is the consummate appeal [219]. A recent study suggested that we might not consider hyaline cartilage as a “final” goal, but as an intermediate stage (Figures 3(e) and 3(f)), and try to stay at this stage. The cells go through the hyaline cartilage stage before forming bone tissue [220]. Researchers used bone morphogenetic protein 2 (BMP2) to initiate the bone formation process after MF and then used an antagonist (VEGFR1) to block the vascular endothelial growth factor, thereby stopping the bone formation process and leaving the new tissue in the hyaline cartilage stage [221].

In summary, the articular cartilage regeneration strategy involving stem cells has achieved encouraging results. The joint cooperation of practitioners from multiple disciplines and fields will help overcome current challenges, and the change in thinking style may open up new strategies for articular cartilage regeneration.

## Figures and Tables

**Table 1:** 

Cell types	References	Animal model	Carrier/scaffold material/delivery method	Groups	Cell source	Single dose	Transplant number	Time point (W)	Conclusion
BMSCs	Lang Li [70]	Canines (*n* = 24) Full-thickness cartilage defects	HA Intra-articular injection	(1) HA (2) BMSCs+HA (3) Control: saline	Allogeneic	1 × 10^7^	1	28	BMSCs+HA can regenerate articular cartilage better than HA alone.
	Wu et al. [71]	Rabbits (*n* = 24) Osteochondral defect	PBS Intra-articular injection	(1) BMSCs (2) PRFr (3) BMSCs+PRFr (4) Control: untreated	Autologous	3 × 10^6^	2	12	The BMSCs+PRFr group had better results in histological evaluation and GAG production.
	Barrachina et al. [72]	Equine (*n* = 18) Chemically induced OA	LRS Intra-articular injection	(1) MSCs-primed+LRS (2) MSCs-naïve+LRS (3) Control: LRS	Allogeneic	1 × 10^7^	2	8 and 24	MSCs-primed+LRS improved clinical symptoms and reduced synovial inflammation, but there was no significant difference from the control group.
	Vayas et al. [73]	Rabbits (*n* = 36) Full-thickness cartilage defects	PLGA microspheres dispersed in a pluronic F-127 solution Intra-articular injection	(1) MF (2) MSCs (3) BMP (3) (4) MF-BMP (3) (5) MSCs-BMP (3) (6) BMP (12) (7) MF-BMP (12) (8) MSCs-BMP (12) (9) Control: untreated	Allogeneic	2.5 × 10^5^	1	12 and 24	Compared with MF, BMP-2 and MSCs repaired articular cartilage defects better and were less invasive.
	Xia et al. [66]	Pigs (*n* = 6) Bilateral medial meniscectomy-induced OA	SPIO nanoparticles Intra-articular injection	(1) SPIO-BMSCs (2) Control: untreated	Allogenic	1 × 10^7^	4	11	The treatment effect of the MSC group was not significantly different from that of the control group.
	Jiang et al. [74]	Rats (*n* = 60) Cartilage defect	PCL-PTHF Cell-scaffold construct implantation	(1) PCL-PTHF with rat tail-derived collagen nanofibers+BMSCs (2) PCL-PTHF with chondroitin sulfate nanofibers+BMSCs (3) Control: untreated	Allogenic	3.14 × 10^5^	1	4 and 8	The PCL-PTHF with rat tail-derived collagen nanofiber group showed better chondrogenesis potential *in vitro* and *in vivo*.
ADSCs	Kohli et al. [75]	Athymic nude rats (*n* = 15) Osteochondral defect	Alpha Chondro Shield Cell-scaffold construct implantation	(1) PA MSCs+Alpha Chondro Shield (2) CD271^+^ MSCs+Alpha Chondro Shield (3) Control: Alpha Chondro Shield	Xenogeneic (human)	5 × 10^4^	1	3	CD271+ADSCs had a stronger ability to promote cartilage regeneration *in vivo*.
	Li et al. [55]	Rabbits (*n* = 60) Cartilage defect	AECM scaffold Cell-scaffold construct implantation	(1) ADSCs+scaffold (2) CD146^+^ ADSCs+scaffold (3) Scaffold (4) Positive control: sham-operated (5) Negative control: untreated	Xenogeneic (human)	5 × 10^5^	1	12 and 24	CD146+ ADSCs promoted better cartilage regeneration than that in the control group.
	Mei et al. [76]	Rats (*n* = 60) ACLT-induced OA	PBS Intra-articular injection	(1) ADSCs+PBS (2) Control: PBS	Allogenic	1 × 10^6^	1	8 and 12	ADSCs had anti-inflammatory effects and inhibited articular cartilage degeneration.
	Critchley et al. [77]	Caprine (*n* = 14) Osteochondral defect	3D-printed PCL alginate hydrogel biphasic scaffold Cell-scaffold construct implantation	(1) The biphasic constructs (2) Maioregen scaffolds (Finceramica)	Allogenic	3 × 10^6^ ADSCss+1 × 10^6^ chondrocytes	1	24	The biphasic constructs regenerated hyaline cartilage *in vivo*.
SMSCs/SFMSCs	Yan et al. [78]	Mice (*n* = 20) Bovine type II collagen-induced OA	PBS Intra-articular injection	(1) SMSCs+PBS (2) Control: PBS	Xenogeneic (human)	1 × 10^6^	3	10	SMSCs prevented arthritis development and suppressed immune responses.
	Kondo et al. [79]	Pigs (*n* = 13) Full-thickness osteochondral defects	No carrier or scaffold MSC aggregate implantation	(1) MSC aggregates (2) Control: untreated	Autologous	4 × 10^6^	1	4 and 12	Autologous synovial MSC aggregates promoted articular cartilage regeneration *in vivo*.
	Neybecker et al. [80]	Nude rats (*n* = 48) ACLT-induced OA	Saline Intra-articular injection	(1) SFMSCs+ saline (2) Saline (3) Control: sham + saline	Xenogeneic (human)	1 × 10^6^	2	4 and 8	Xenogenic SFMSCs exerted neither chondroprotection nor inflammation in ACLT-induced OA.
	Li et al. [81]	Rats (*n* = 30) Full-thickness cartilage defects	Hyperbranched poly-PEGDA/HA hydrogel Injected into the cartilage defect site	(1) AFF-MSCs/hydrogel (2) Hydrogel (3) Control: PBS	Xenogeneic (human)	1 × 10^6^	1	4 and 8	The composite material significantly repaired articular cartilage defects.
UCBMSCs/WJMSCs	Zhang et al. [6]	Goats (*n* = 6) Full-thickness cartilage defects	AECM scaffold Cell-scaffold construct implantation	(1) MSCs+ scaffold (2) MF	Xenogeneic (human)	1 × 10^6^	1	24 and 36	The cell-scaffold constructs maintained the integrity of subchondral bone and regenerated hyaline cartilage.
	Liu et al. [82]	Rabbits (*n* = NS) Full-thickness cartilage defects	ECM scaffold Cell-scaffold construct implantation	(1) hWJMSCs-scaffold (2) hWJMSCs-C-scaffold (3) Scaffold (4) Control: untreated	Xenogeneic (human)	NS	1	12, 24, 28, and 64	WJMSC composite ECM scaffold regenerated hyaline cartilage *in vivo*. Undifferentiated WJMSCs had a better repair effect.
	Xing et al. [83]	Rats (*n* = 18) ACLT and medial meniscectomy-induced OA	HA Intra-articular injection	(1) HA+MSCs (2) HA (3) Control: saline	Xenogeneic (human)	1 × 10^6^	1	6 and 12	At 6 weeks, the therapeutic effect of the HA+MSCs group was significantly better than that of other groups, but there was no significant difference between the groups at 12 weeks.
iPSCs	Rim et al. [84]	Rats (*n* = NS) Full-thickness cartilage defects	hiChondroPellet group: no carrier or scaffold Transplant directly to the defect site hiChondrocytes group: PBS Injected into the cartilage defect site	(1) hiChondroPellet (2) hiChondrocytes+PBS (3) Defect control: untreated (4) Normal control	Xenogeneic (human)	hiChondroPellets or 1 × 10^6^ hiChondrocytes	1	8	Both the chondropellets and the chondrocytes derived from iPSCs had therapeutic effects on osteochondral defects.
	Kotaka et al. [25]	Nude rats (*n* = 54) Full-thickness cartilage defects	Atelocollagen Transplant directly to the defect site by an external magnetic field	(1) Magnetic force+iPS+ atelocollagen (2) iPS+ atelocollagen (3) Control: atelocollagen	Xenogeneic (human)	1 × 10^5^	1	4, 6, and 8	The histological score of the treatment group was significantly better than that of the control group.
ESCs/EMSCs	Jiang et al. [60]	Rhesus macaques (*n* = 8) Spontaneous OA	Saline Intra-articular injection	(1) EMSCs (2) BMSCs (3) Control: saline	EMSC group: xenogeneic (human) BMSC group: allogeneic	5 × 10^6^	3	4, 8, 12, 16, 24, and 36	The degrees of joint swelling and imaging examination results in the EMSC group and BMSC group were significantly improved.
	Gibson et al. [85]	Nude rats (*n* = 15) Cartilage defects	No carrier or scaffold Transplant MSC pellets directly to the defect site	(1) MSC pellets (untreated) (2) MSC pellets (pretreated with BMP-2 and Wnt5a) (3) Control: empty defects	Xenogeneic (human)	2.5 × 10^5^	1	4 and 8	Cartilage progenitor cell particles derived from hESCs pretreated with BMP-2 and Wnt5a induced hyaline cartilage regeneration *in vivo*.

**Table 2:** 

Cell types	References	Cell source	Single dose	Transplant number	K-L grade	Age	Sample size	Carrier/scaffold material	Follow-up (M)	Conclusion
BMSCs/BMACs	Chahal et al. [86]	Autologous	1 × 10^6^ 1 × 10^7^ 5 × 10^7^	1	III-IV	40-65	12	Excipient	12	The clinical symptoms significantly improved in the 5 × 10^7^ cell group.
	Shapiro et al. [87]	Autologous	5 mL BMAC (1.7 × 10^5^ cells)	1	I-III	42-68	25	Platelet-poor bone marrow plasma	12	BMACs relieved pain caused by OA. However, at 12 months, BMACs had no significant advantage compared with saline.
	Emadedin et al. [88]	Autologous	4 × 10^7^	1	II-IV	18-65	43	Saline +2% human serum albumin	6	BMSCs significantly relieved the pain of patients with OA.
	Shadmanfar et al. [89]	Autologous	4 × 10^7^	1	II-IV	18-65	30	Saline	12	BMSCs alleviated clinical symptoms, but their efficacy at 12 months was not significantly different from that of the placebo.
ADSCs	Kim and Koh [61]	Autologous	4.26 × 10^6^	1	III-IV	53-65	100	NS	At least 36	HTO combined with ADSCs improved IKDC and Lysholm scores in patients with OA.
	Song et al. [65]	Autologous	1 × 10^7^ 2 × 10^7^ 5 × 10^7^	3	≥II	40-70	14	NS	24	Autologous ADSCs were safe and significantly improved the symptoms of OA. The effect of repeated injections of high-dose cells was more obvious.
	Kim et al. [67]	Autologous	4.7 × 10^6^	1	III-IV	42-68	70	Allogenic cartilage (MegaCartilage) or fibrin glue (Greenplast kit®)	27.6	HTO+autologous MSCs+allogeneic cartilage implantation more effectively treated OA.
UCBMSCs/WJMSCs	Sadlik et al. [68]	Allogenic	NS	1	NS	NS	5	Porcine type I/II collagen matrix scaffold (ChondrO-Gide)	12	WJMSCs can be used to induce articular cartilage regeneration.
	Matas et al. [90]	Allogenic	2 × 10^7^	2	I-III	40-65	26	Saline with 5% AB plasma	13	Repeated injection of WJMSCs was safe and significantly improved the clinical symptoms of OA.
	Song et al. [62]	Allogenic	2.5 × 10^6^ cells/cm^2^	1	I-III	>40	128	4% HA (CARTISTEM®)	24	Allogeneic UCBMSCs significantly reduced the pain of OA joints and improved joint function.
ESCs or PLMSCs	Khalifeh Soltani et al. [91]	Allogenic	5 − 6 × 10^7^	1	II-IV	35-75	20	NS	6	Allogeneic PLMSCs relieved the symptoms of OA joints.

BMAC: bone marrow aspiration and concentration; SMSCs/SFMSCs: synovial-derived mesenchymal stem cells/synovial fluid-derived mesenchymal stem cells; UCBMSCs/WJMSCs: umbilical cord blood-derived mesenchymal stem cells/umbilical cord Wharton's jelly derived mesenchymal stem cells; iPSCs: induced pluripotent stem cells; ESCs/EMSCs: embryonic stem cells/ESC-derived mesenchymal stem cells; LRS: lactate's ringer solution; MF: microfracture; SPIO: superparamagnetic iron oxide; PAMSCs: plastic adherent MSCs; AECM: articular cartilage extracellular matrix; HTO: high tibial osteotomy; RA: rheumatoid arthritis; WOMAC: the Western Ontario and McMaster Universities Arthritis Index; VAS: visual analog scale; PLMSCs: placenta-derived MSCs; PCL-PTHF: electrospun nanofibers composed of cartilage matrix components (collagen or chondroitin sulfate) and poly(-caprolactone)-polytetrahydrofuran; NS: not specified; PRFr: platelet-rich fibrin releasate; K-L grade: Kellgrene-Lawrence grade.
